# Inclusion Effect of Various Levels of Jack Mackerel Meal in Olive Flounder (*Paralichthys olivaceus*) Diets Substituting 50% Fish Meal with Duck By-Product Meal on Growth and Feed Utilization

**DOI:** 10.3390/ani14152184

**Published:** 2024-07-26

**Authors:** Md Rabiul Islam, Sung Hwoan Cho, Taeho Kim

**Affiliations:** 1 Division of Convergence Interdisciplinary Education of Maritime and Ocean Contents, Korea Maritime and Ocean University, Busan 49112, Republic of Korea; rabiul.islam@bsmrau.edu.bd; 2 Department of Aquaculture, Bangabandhu Sheikh Mujibur Rahman Agricultural University, Gazipur 1706, Bangladesh; 3 Division of Convergence on Marine Science, Korea Maritime and Ocean University, Busan 49112, Republic of Korea; 4 Department of Marine Production Management, Chonnam National University, Yeosu 59626, Republic of Korea; kimth@chonnam.ac.kr

**Keywords:** fish meal replacement, jack mackerel meal, feed utilization, *Paralichthys olivaceus*, economic profit index

## Abstract

**Simple Summary:**

Fish meal (FM) is considered the primary protein source in fish feed, but the most expensive ingredient, which ultimately increases feed cost. Application of a substitute for FM in fish feed can lower feed cost, but it commonly leads to reduced palatability and lowered feed consumption of fish. Jack mackerel meal (JMM) has been reported to be an effective attractant and/or stimulant in the olive flounder diet. This study revealed that inclusion of 50% jack mackerel meal (JMM) at the expense of FM in the olive flounder (*Paralichthys olivaceus*) diet replacing 50% FM with duck by-product meal (DBM) lowered feed cost, but improved feed consumption and growth performance and economic return to the farmer. The results of this study will help olive flounder producers to improve their profitability.

**Abstract:**

This experiment was performed to evaluate the inclusion impact of various levels of jack mackerel meal (JMM) in olive flounder (*P. olivaceus*) feeds substituting 50% FM by duck by-product meal (DBM) on growth, feed availability, and economic efficiency. Seven experimental diets were prepared. The control (Con) diet contained 60% FM. Fifty percent FM in the Con diet was substituted with DBM, and then the graded levels (0%, 10%, 20%, 30%, 40%, and 50%) of JMM were added instead of FM, named the DJ0, DJ10, DJ20, DJ30, DJ40, and DJ50 diets, respectively. All feeds were assigned to triplicate fish groups. At the end of 56 days’ feeding, fish fed the DJ40 and DJ50 diets exhibited comparable weight gain and specific growth rate to fish fed the Con diet. Higher feed consumption was observed in fish fed the Con, DJ40, and DJ50 diets compared to fish fed the DJ0 and DJ10 diets. Lower feed conversion ratio was observed in fish fed the Con diet compared to fish fed the DJ0, DJ10, DJ20, and DJ30 diets. Furthermore, the DJ50 diet led to the highest economic profit index (EPI). In conclusion, inclusion of 50% JMM in the olive flounder diet replacing 50% FM with DBM seems to be the most recommendable dietary treatment based on growth and feed consumption of olive flounder and EPI.

## 1. Introduction

The Republic of Korea is one of the leading aquacultural countries globally [1]. Marine finfish production in South Korea is dominated by olive flounder (*Paralichthys olivaceus*), and contributed to an annual production of 45,801 metric tons and an economic value of USD 386 million in 2022 [2]. In a land-based intensive olive flounder production system, the widespread use of raw fish-based moist pellets (MP) results in high production cost, nutrient loss, water pollution, disease outbreak, and mortality [3]. The utilization of formulated feed (FF) including extruded pellets has proven to be more environmentally-friend than MP, leading to elevated growth and nutrient utilization of fish [4]. However, FF for olive flounder relies heavily on fish meal (FM), containing up to 60% of the primary protein source [5]. The increasing demand and decreasing production of FM over time have contributed to rising its cost, prompting scientists to look for an alternative to FM in aquafeeds [6].

Various plant protein sources, including microalgae [7], macroalgae [8], dried grain from rice distillers [9], and soybean and cottonseed meal [10], have been explored as replacements for FM in olive flounder diets because of their sufficient protein content, affordable price, and year-round availability. However, challenges such as high fiber content, amino acid (AA) imbalances, and the existence of antinutritional factors commonly limit the extensive application of plant proteins in fish diets because of reduced palatability and feed consumption and compromised growth rate of fish [11,12]. Therefore, animal protein sources are favored over plant protein sources in fish diets due to their abundant AA and fatty acid (FA) profiles [13]. It has been reported that 10–50% FM can be replaced with alternative animal protein sources, such as silkworm pupae meal [14], chicken by-product meal [15], meat meal [16], meat and bone meal, and tuna by-product meal [17], without undesirable impacts on the growth or feed utilization of olive flounder.

Duck by-product meal (DBM) can be considered a prospective alternative for FM in fish feed. DBM is clean, dried, and ground duck tissue including skin, bone, head, feet, feathers, and blood sourced from duck processing plants where ducks are slaughtered for human consumption [18]. In 2021, global production of duck meat was 6.2 million metric tons. South Korea is considered one of the top duck meat producers, with production of 74,968 metric tons [19]. During duck processing, several thousand metric tons of organic by-products are being produced from the processing plant. DBM, an inexpensive ingredient but rich in protein and lipid, shows a high possibility for use as an FM replacement in the olive flounder diet. However, reduced feed palatability and feed consumption are the common concerns when FM is substituted with alternative protein sources in the olive flounder diet [16]. Therefore, inclusion of protein ingredient with feed attractant and stimulant effect in low-FM diets is one of the best methods to resolve those undesirable problems.

Feed attractant and stimulant are usually low-molecular-weight compounds, such as free AA, nucleosides, nucleotides, organic acids, and quaternary ammonium (NH_4_) bases, which are added to feed to enhance palatability [20]. Furthermore, incorporating feed attractants in diet facilitates faster feed ingestion and provides supplemental nutrients for protein and energy metabolism [21]. Both natural and synthetic stimulants are used in fish feed formulations. However, the absence of certain effective components in the synthetic stimulant renders them inferior to natural stimulant [22]. Carr et al. [23] emphasized that the tissue extracts of marine organisms contain natural stimulants and identified low-molecular-weight components from 30 species of marine fish, including jack mackerel (*Trachurus japonicus*), mollusks, and crustaceans. Jack mackerel meal (JMM) has been demonstrated to be a significant attractant and/or stimulant in various fish species, such as olive flounder [24], rockfish (*Sebastes schlegeli*) [25,26], and yellowtail (*Seirola quinqueradiata*) [27]. Furthermore, Ikeda et al. [28] and Takakuwa et al. [29] revealed that AA groups, particularly histidine and nucleotides, and inosine monophosphate (IMP) in the muscle extracts of jack mackerel showed the highest feed stimulant activity on olive flounder and greater amberjack (*Seriola dumerili*), respectively. Kim et al. [30] also revealed that among 16 protein ingredients, JMM showed the strongest feed attractiveness to rockfish. Inclusion of JMM in formulating low-FM diets can be a sustainable fish culture technique in increasing feed consumption and growth performance of fish.

Therefore, the present experiment was performed to elucidate the inclusion effect of graded levels of JMM in the low-FM diets of olive flounder replacing 50% FM with DBM on the growth and feed availability of olive flounder and to assess economic efficiency.

## 2. Materials and Methods

### 2.1. Experimental Diet Preparation

Seven diets with isonitrogenous content of 52.0% and isolipidic of 13.5% were prepared (Table 1). The primary protein sources in the control (Con) diet were FM (60%) and fermented soybean meal (10%). In addition, wheat flower (22.5%), and each of fish and soybean oil (2.5%) were used as the carbohydrate and lipid source, respectively, in the Con diet. Fifty percent FM in the Con diet was substituted with DBM and then the graded levels (0%, 10%, 20%, 30%, 40%, and 50%) of JMM were included at the coast of FM, referred as the DJ0, DJ10, DJ20, DJ30, DJ40, and DJ50 diets, respectively. After thoroughly mixing the ingredients of each diet, water was added at a ratio of 3:1 to form a dough. After considering the mouth size of olive flounder, the dough was then pelletized (4−6 mm in diameter) using a laboratory extruder. Finally, after drying at 30 °C in a forced-air oven for 48 h, all the experimental diets were stored at −20 °C until further use.

### 2.2. Experimental Conditions

Healthy juvenile olive flounder of similar sizes were bought from a private fish farm and transported to the laboratory. Prior to the feeding experiment, all the fish were acclimatized to the rearing conditions for 2 weeks by providing a commercial pellet twice a day at a biomass ratio of 1.5–3%. Total of 525 juvenile fish (initial weight of 20.27 ± 0.03 g; mean ± SEM) were randomly distributed into 21 50 L flow-through tanks (53.4 × 34.0 × 27.4 cm) (25 fish/tank). Fifteen fish were stocked into each tank, and then the remaining ten fish were added to adjust to the same initial total weight of fish per tank. Each tank received sand-filtered seawater at a flow rate of 4.2 L/min and continuous aeration. A multifunctional water quality meter (AZ-8603, AZ Instrument, Taichung city, China) was used daily to monitor water quality. The temperature, dissolved oxygen, salinity, and pH ranged from 16.3 to 21.6 °C (19.7 °C ± 1.59 °C; mean ± SD), 7.3–8.0 mg/L (7.5 ± 0.22 mg/L), 30.8–32.2 g/L (31.3 ± 0.38 g/L), and 7.4–7.7 (7.5 ± 0.10), respectively.

Each formulated diet was assigned to triplicated groups of fish. Throughout the 56-day feeding trial, olive flounder were carefully hand-fed to apparent satiation twice a day (08:30 and 17:30). The bottoms of the tanks were cleaned by siphoning daily after feeding in the morning, and the photoperiod followed the natural cycle. A daily feed supply to each tank was recorded and uneaten feed was not collected. Dead fish were removed immediately upon discovery and weighed. The feeding trial and subsequent handling and sampling of experimental fish were carried out as per the ethical guidelines of the Korea Maritime and Ocean University.

### 2.3. Measurement of the Biological Indices of Fish

After the 56-day feeding trial, all surviving fish were anesthetized with MS-222 at a concentration of 100 ppm, followed by 24 h starvation. All live fish from each tank were counted to calculate the survival rate and their collective weight was measured to determine weight gain. Ten randomly selected anesthetized fish from each tank were individually weighed, measured in total length, and then dissected to collect the viscera and liver for calculating the viscerosomatic index (VSI) and hepatosomatic index (HSI). The growth performance, feed utilization, and biological indices of olive flounder were calculated as the following [31]: specific growth rate (SGR, %/day) = (Ln final weight of fish − Ln initial weight of fish) × 100/days of feeding (56 days), feed conversion ratio (FCR) = feed supplied/weight gain of fish, protein efficiency ratio (PER) = weight gain of fish/protein supplied, protein retention (PR, %) = (final body protein − initial body protein) × 100/protein supplied, condition factor (K, g/cm^3^) = body weight of fish (g) × 100/total length of fish (cm)^3^, VSI (%) = viscera weight of fish × 100/body weight of fish, and HSI (%) = liver weight of fish × 100/body weight of fish.

### 2.4. Measurements of the Biochemical Composition of the Samples

Ten fish at the beginning of the trial and ten fish from each tank after the measurements of biological indices were homogenized and used for the proximate composition analysis. Chemical analyses for the experimental feeds and fish were performed according to the standard AOAC [32] method. Crude protein content was determined by a Kjeldahl apparatus (Kjeltec 2100 Distillation Unit, Foss Tecator, Hoganas, Sweden), and crude lipid content was determined by ether-extraction method (Soxtec TM 2043 Fat Extraction System, Foss Tecator, Hoganas, Sweden). Moisture content was determined by oven-drying at 105 °C for 24 h, and ash content was determined by using a muffle furnace at 550 °C for 4 h. To analyze AA, excluding methionine, cysteine, and tryptophan, the experimental feeds and whole-body fish were hydrolyzed with 6 N HCl for 24 h at 110 °C followed by ion exchange chromatography with an AA analyzer (L-8800 Auto-analyzer: Hitachi, Tokyo, Japan). To measure methionine and cysteine content, the samples were oxidized with performic acid at below 5 °C for 24 h to obtain methionine sulfone and cysteic acid, and they were then freeze-dried twice with deionized water. Then, the freeze-dried samples were hydrolysed and analyzed following similar process used for the other amino acids. Tryptophan analysis was conducted using high-performance liquid chromatography (S1125 HPLC pump system; Sykam GmbH, Eresing, Germany). Lipids for FA analyses in the feeds and whole-body fish were extracted using a mixture of chloroform and methanol (2:1 *v*/*v*), following the method of Folch et al. [33]. FA methyl esters were prepared by transesterification with 14% BF_3_-MeOH and analyzed by gas chromatography (Trace GC, Thermo, Waltham, MA, USA).

### 2.5. Analysis of Plasma and Serum Measurements of Fish

Blood was drawn from the caudal veins of five anesthetized fish from each tank using heparinized syringes. The plasma was then extracted and kept in separate aliquots in a freezer at −70 °C after centrifugation (2716× *g* at 4 °C) for 10 min. An automated chemistry system (Fuji Dri-Chem NX500i, Fujifilm, Tokyo, Japan) was utilized to analyze aspartate transaminase (AST), alanine transaminase (ALT), alkaline phosphatase (ALP), total bilirubin (T-BIL), total cholesterol (T-CHO), total protein (TP), triglyceride (TG), and albumin (ALB). Plasma samples of fish from each tank were pooled.

In addition, blood was drawn from five anesthetized fish from each tank using syringes. The serum was extracted and kept in separate aliquots in a freezer at −70 °C after centrifugation (2716× *g* at 4 °C) for 10 min. Serum lysozyme activity was measured using the turbidimetric assay as per Lange et al. [34], and superoxide dismutase (SOD) was measured using a commercial SOD Assay kit (Sigma MBS705758; Sigma, St. Louis, MO, USA) according to the manufacturer’s instructions.

### 2.6. Economic Analysis of the Study

The economic assessment of the experiment was performed by applying the formula proposed by Martínez-Llorens et al. [35]: economic conversion ratio (ECR, USD/kg) = feed consumption of fish (kg) × feed cost (USD/kg)/weight gain (kg), and economic profit index (EPI, USD/fish) = (final weight of fish (kg/fish) × selling price of fish (USD/kg)) − (feed consumption of fish (kg) × diet price (USD/kg)). The cost per kilogram (USD/kg) for each ingredient was as follows: FM = 2.23, DBM = 0.60, JMM = 2.67, fermented soybean meal = 0.70, wheat flour = 0.55, fish oil = 2.76, soybean oil = 1.79, vitamin premix = 8.28, mineral premix = 6.66, and choline = 1.30. The selling price of olive flounder was assumed as USD 12.44 /kg.

### 2.7. Statistical Analysis

Significant differences in means were examined using one-way ANOVA and Tukey’s post hoc test after the normality (Shapiro–Wilk) and homogeneity (Levene) tests on SPSS version 24.0 (SPSS Inc., Chicago, IL, USA). Percentage data underwent arcsine transformation prior to statistical analysis. Additionally, a follow-up trend analysis using orthogonal polynomial contrasts excluding the Con diet was conducted to evaluate whether the effect demonstrated linear, quadratic, or cubic trends. Statistical significance level was set at *p* < 0.05. Furthermore, regression analysis was undertaken to identify the best-fitting model.

## 3. Results

### 3.1. AA and FA Profiles of the Feeds

All essential AAs (EAAs) except for methionine and total content of EAA (∑EAA) in FM were relatively low compared to those in JMM, but higher than those in DBM, except for arginine content (Table 2). Leucine and lysine as well as aspartic acid and glutamic acid were the richest EAA and NEAA, respectively, in all FM, JMM, and DBM. Increased inclusion levels of JMM in the low-FM diets tended to increase all EAA content, except for phenylalanine.

FM exhibited higher total content of saturated fatty acid (∑SFA), but lower total content of monounsaturated FA (∑MUFA) and lower total content of n-3 highly unsaturated FA (∑n-3 HUFA) including eicosapentaenoic acid (EPA, C20:5n-3) and docosahexaenoic acid (DHA, C22:6n-3) compared to JMM (Table 3). However, DBM exhibited higher ∑MUFA compared to both FM and JMM, but lower for EPA, DHA, and ∑n-3 HUFA (Table 3). Notably, elevated inclusion levels of JMM in the low-FM diets substituting 50% FM with DBM led to decreased ∑SFA, but increased ∑MUFA and ∑n-3 HUFA.

### 3.2. Growth and Feed Availability of Fish

**Figure 1:**
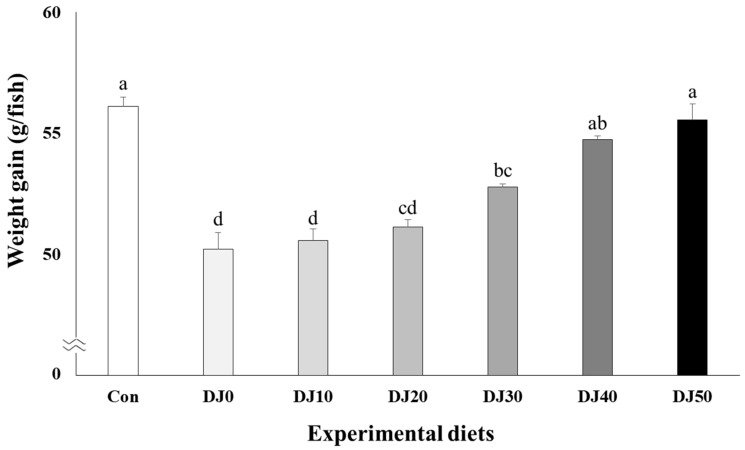
Weight gain (g/fish) of olive flounder (*Paralichthys olivaceus*) fed the experimental diets for 56 days (mean of triplicate ± SE) (*p* < 0.001). Con: 60% FM; DJ0–DJ50: 50% DBM with 0% to 50% JMM. (Orthogonal polynomial contrast (linear; *p* = 0.001, quadratic; *p* = 0.080, cubic; *p* = 0.156); Y = 1.166689X + 48.4182, *p* < 0.001, R^2^ = 0.8524).

**Figure 2:**
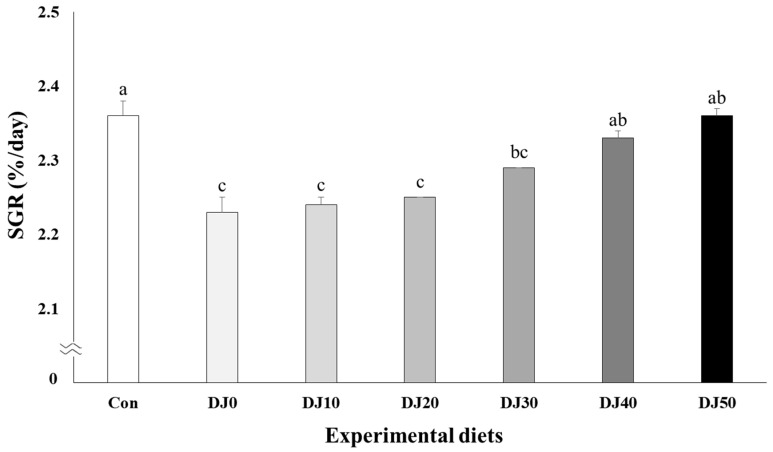
Specific growth rate (SGR, %/day) of olive flounder (*Paralichthys olivaceus*) fed the experimental diets for 56 days (mean of triplicate ± SE) (*p* < 0.001). Con: 60% FM; DJ0–DJ50: 50% DBM with 0% to 50% JMM. (Orthogonal polynomial contrast (linear; *p* = 0.001, quadratic; *p* = 0.177, cubic; *p* = 0.334); Y = 0.027709X + 2.1858, *p* < 0.001, R^2^ = 0.8237).

Survival of fish varied between 94.67% and 97.33% and was not significantly (*p* > 0.05) changed by dietary treatments (Table 4). Weight gain increased in fish fed the Con and DJ50 diets compared to fish fed the DJ0, DJ10, DJ20, and DJ30 diets, but was comparable to fish fed the DJ40 diet (Figure 1) (*p* < 0.001). Accordingly, SGR increased in fish fed the Con diet when compared to fish fed the DJ0, DJ10, DJ20, and DJ30 diets, but was comparable to fish fed the DJ40 and DJ50 diets (Figure 2) (*p* < 0.001). In orthogonal polynomial contrast, significant linear relationships (*p* = 0.001 for both) were observed in weight gain and SGR of olive flounder and inclusion levels of JMM in the low-FM diets. Regression analysis indicated linear relationships as the best-fit models between inclusion levels of JMM in the low-FM duets replacing 50% FM by DBM and weight gain (Y = 1.166689X + 48.4182, *p* < 0.001, R^2^ = 0.8524) and SGR (Y = 0.027709X + 2.1858, *p* < 0.001, R^2^ = 0.8237), respectively.

**Figure 3:**
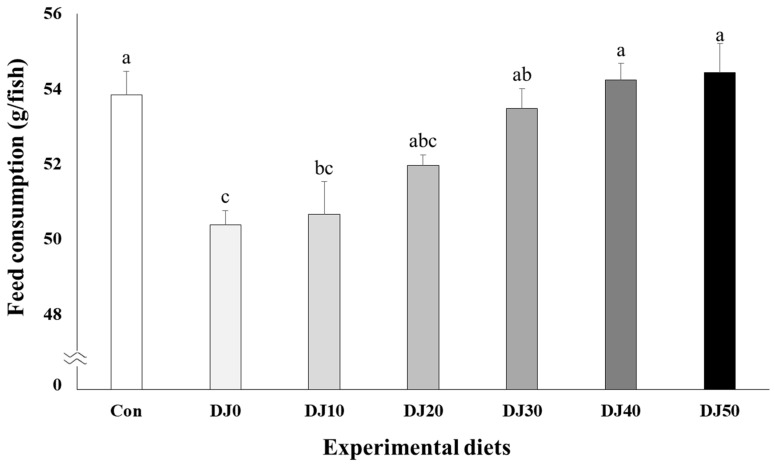
Feed consumption (g/fish) of olive flounder (*Paralichthys olivaceus*) fed the experimental diets for 56 days (mean of triplicate ± SE) (*p* < 0.001). Con: 60% FM; DJ0–DJ50: 50% DBM with 0% to 50% JMM. (Orthogonal polynomial contrast (linear; *p* = 0.001, quadratic; *p* = 0.638, cubic; *p* = 0.195); Y = 0.929921X + 49.2673, *p* < 0.001, R^2^ = 0.7368).

Higher feed consumption was observed in fish fed the Con, DJ40, and DJ50 diets compared to olive flounder fed the DJ0 and DJ10 diets, but comparable to fish fed the DJ20 and DJ30 diets (Figure 3) (*p* < 0.001). In orthogonal polynomial contrast, a significant linear relationship (*p* = 0.001) was observed in feed consumption and inclusion levels of JMM in the low-FM feeds. Regression analysis indicated the linear model was the best fit between inclusion levels of JMM in the low-FM feeds and feed consumption of fish (Y = 0.929921X + 49.2673, *p* < 0.0001, R^2^ = 0.7368).

Significantly a greater FCR was found in fish fed the Con diet compared to olive flounder fed the DJ0, DJ10, DJ20, and DJ30 diets (*p* < 0.003), but comparable to olive flounder fed the DJ40 and DJ50 diets. In orthogonal polynomial contrast, significant linear (*p* = 0.050) and quadratic (*p* = 0.026) relationships were observed in FCR and inclusion levels of JMM in the low-FM feeds. Regression analysis indicated the quadratic model (*p* < 0.012 and adjusted R^2^ = 0.368) to be the best fit between inclusion levels of JMM in the low-FM feeds and FCR. PER of olive flounder fed the DJ50 diet was significantly (*p* < 0.005) higher than that of olive flounder fed the DJ0 and DJ10 diets, but not significantly (*p* > 0.05) different from that of olive flounder fed the Con, DJ20, DJ30, or DJ40 diets. In orthogonal polynomial contrast, a significant linear relationship (*p* = 0.001) was observed in PER and inclusion levels of JMM in the low-FM feeds. Regression analysis indicated a linear model (*p* < 0.001 and adjusted R^2^ = 0.533) to be the best fit between inclusion levels of JMM in the low-FM feeds and PER. PR of fish was not significantly (*p* > 0.142) changed by dietary treatments.

However, K of fish varied from 0.84 to 0.91 g/cm^3^, VSI varied from 4.14% to 4.63%, and HIS varied from 1.09% to 1.43%, but these values were not significantly (*p* > 0.05 for all) affected by dietary treatments.

### 3.3. Proximate Composition of the Whole-Body Fish

Moisture, crude protein, crude lipid, and ash content of the whole-body fish ranged from 74.63% to 75.63%, 16.10% to 17.47%, 2.80% to 3.70%, and 3.27% to 4.47%, respectively (Table 5). None of these measurements was significantly (*p* > 0.05 for all) influenced by dietary treatments.

### 3.4. Plasma and Serum Measurements of Fish

Plasma AST, ALT, ALP, T-BIL, T-CHO, TG, TP, and ALB level of fish varied from 8.33 to 10.67 U/L, 4.67 to 6.00 U/L, 70.00 to 73.67 U/L, 0.13 to 0.23 mg/dL, 179.00 to 181.67 mg/dL, 438.3 to 443.33 mg/dL, 1.73 to 2.33 g/dL, and 0.43 to 0.63 g/dL, respectively (Table 6). The experimental diets did not (*p* > 0.05 for all) alter these parameters. Serum SOD ranged from 2.57 to 3.12 ng/L and lysozyme activity of fish ranged from 296.67 to 546.67 U/mL. The experimental diets did not significantly (*p* > 0.815 and *p* > 0.878, respectively) affect the serum SOD or lysozyme activity of fish.

### 3.5. AA and FA Profiles of the Whole-Body Fish

The experimental diets did not show any significant impact on the AA profiles of the whole-body olive flounder (Table 7) (*p* > 0.05).

However, significantly higher ∑SFA, EPA, DHA, and ∑n-3 HUFA content were found in the whole-body olive flounder fed the Con feed than those of olive flounder fed all other feeds (Table 8) (*p* < 0.001 for all). In orthogonal polynomial contrast, significant linear (*p* = 0.002 and *p* = 0.001, respectively) relationships were observed in the ∑SFA and ∑MUFA in the whole-body olive flounder and inclusion levels of JMM in the low-FM diets. However, significant linear (*p* = 0.000), quadratic (*p* = 0.001), and cubic (*p* = 0.001) relationships were observed in the ∑n-3 HUFA in the whole-body of olive fish and inclusion levels of JMM in the low-FM diets. In regression analysis, the linear model (*p* < 0.001; adjusted R^2^ = 0.927 and *p* < 0.003; adjusted R^2^ = 0.891, respectively) was found to be the best fit model between inclusion levels of JMM in the low-FM diets and the ∑SFA and ∑MUFA of olive flounder and. However, the cubic (*p* < 0.001 and adjusted R^2^ = 0.964) model was found to be the best fit model between inclusion levels of JMM in the low-FM diets and the ∑n-3 HUFA of fish in regression analysis.

### 3.6. Economic Analysis of the Experiment

The prices of low-FM diets increased with JMM inclusion levels, but their prices were all still much lower than that of the Con diet (Table 9). The ECR of the Con diet was significantly (*p* < 0.001) higher than that of all FM-replaced feeds. However, no significant (*p* > 0.05) difference in ECR was found among the DJ20, DJ30, DJ40, and DJ50 diets. In orthogonal polynomial contrast, significant linear (*p* = 0.001) and quadratic (*p* = 0.030) relationships were observed in ECR and inclusion levels of JMM in the low-FM feeds. In regression analysis, the quadratic model (*p* < 0.001 and adjusted R^2^ = 0.732) was found to be the best fit between inclusion levels of JMM in the low-FM feeds and ECR. Superior (*p* < 0.001) EPI was obtained in the DJ40 and TJ50 diets compared to the DJ0, DJ10, DJ20, and DJ30 diets, but comparable to the Con diet. In orthogonal polynomial contrast, significant linear (*p* = 0.000) and quadratic (*p* = 0.045) relationships were observed in EPI and inclusion levels of JMM in the low-FM feeds. In regression analysis, the linear model (*P* < 0.001 and adjusted R^2^ = 0.812) was found to be the best fit between inclusion levels of JMM in the low-FM feeds and EPI.

## 4. Discussion

No significant differences in weight gain or SGR of olive flounder fed the DJ40 and DJ50 diets compared to fish fed the Con diet in this experiment implied that 50% of FM could be replaced with DBM without negatively affecting the growth performance of fish, as long as 40–50% of JMM is included at the expense of FM in a 60% FM-based diet. Nevertheless, linear increase in weight gain and SGR of fish with increased JMM inclusion levels in low-FM diets in regression analysis indicated that the DJ50 diet appeared to be the most recommendable feeding strategy according to the growth performance of olive flounder. Furthermore, inferior ECR in all DJ diets compared to the 60% FM-based diet appeared to be more feasible than the Con diet in this experiment because of the lower price of DBM than FM. In particular, the highest EPI, representing the greatest economic return to the farmer, was observed in the DJ50 diet based on the economic analysis (Table 9). This also supports the finding of this study that the DJ50 diet was the most desirable dietary treatment based on the results of weight gain and SGR of fish in regression analysis. However, inferior weight gain and SGR of olive flounder fed the DJ0 diet in contrast to olive flounder fed the Con diet implied that 50% FM substitution with DBM in a diet without JMM inclusion could not catch up with the growth of olive flounder fed the 60% FM-basal diet. However, the gradual improvement in growth performance of fish fed the low-FM diets replacing 50% FM by DBM with increased JMM inclusion levels proved that inclusion of JMM in low-FM diets effectively boosted the growth performance.

Enhanced growth performance of fish appeared to be proportional to enhanced feed consumption in all DJ diets in this study. Linear increases in feed consumption of olive flounder fed the low-FM diets with increased JMM inclusion levels were probably because of the feed-enhancing effect of JMM, indicating that 50% JMM inclusion is the most recommended feeding strategy in low-FM diets substituting 50% FM with DBM. This desirable effect might be attributed to the relatively high levels of EAA, except for methionine, and NEAA present in JMM over FM. Likewise, previous studies have also reported an increase in feed consumption of rockfish (*Sebastes schlegeli*) and olive flounder when JMM was introduced as the feed enhancer and/or stimulant in low-FM diets [26,31]. The feeding response of fish is influenced by two primary chemoreception channels: olfaction, being responsible for smell and location, and gustation, being responsible for taste or consumption [41,42]. Some AAs, including lysine, methionine, glycine, alanine, and proline, are the major classes of olfactory and gustatory feeding stimulants for fish [12,20]. Furthermore, the studies performed by Takakuwa et al. [29] and Ikeda et al. [28] pointed out that muscle extracts of jack mackerel are an abundant source of AAs and nucleotides, which exhibit a feeding-stimulatory effect on fish. The AAs and nucleotides possess potent chemosensory capabilities and contribute significant flavor and taste in fish diets [43]. The incorporation of attractants into feed not only enables quicker access to feed but also creates conditions for faster ingestion [20].

FCR tended to decrease however, PER of olive flounder tended to improve with increasing JMM inclusion levels in low-FM diets in this experiment. This finding aligns with Kikuchi’s [44] study, in which the maximum weight gain, FE, and PER were reported in olive flounder fed a low-FM diet supplemented with 5% blue mussel meat as a feed stimulant in a 75% FM-based diet. Tharaka et al. [45] and Khosravi et al. [46] also reported improvements in the growth rate, feed consumption, and PER of olive flounder fed diets incorporated with protein hydrolysates (tilapia, shrimp, and krill hydrolysates) and a low-FM diet supplemented with krill meal, respectively. Contrary to this study, however, increased JMM inclusion as feed stimulant up to 100% in the low-FM diets did not change PER of olive flounder [24,31]. This discrepancy in the impact of JMM inclusion on feed utilization of olive flounder could potentially be attributed to differences in feed formulation, protein sources, nutritional profiles including AAs, and the types and doses of stimulants used.

Somatic indices, such as K, HIS, and VSI, are used to evaluate the health condition of fish [47]. In this experiment, these indices of fish were not changed by dietary treatments. This agrees with Kim et al.’s [48] report, where dietary FM replacement with different animal by-products did not influence these indices in olive flounder. Moreover, supplementation with crude attractants (10% fish soluble, 5% squid meal, 5% krill meal, and their mixture) and marine protein hydrolysates as feed enhancers in low-FM diets had no impacts on K, VSI, or HSI of red sea bream (*Pagrus major*) [49,50].

AAs are essential precursors of vital biomolecules (antibodies, enzymes, hormones, and nucleotides), and deficiencies in EAAs might affect fish growth, feed utilization, immunity, survivability, and many other physiological process [39]. Therefore, the AA profiles of a diet are highly crucial in preparing low-FM feeds. The requirements for arginine (2.04–2.10%), lysine (1.55–1.97%), and threonine (1.03%) for olive flounder were met in all formulated feeds in the present experiment [36,37,39]. However, the methionine content in all experimental feeds including the Con diet seemed to be slightly lower than the requirement (1.44–1.49%) in the presence of 0.06% cysteine for olive flounder [38]. Since cysteine can spare the methionine requirement in fish diets, which has been reported to be 50% and 60% in the diets of red drum (*Sciaenops ocellatus*) and channel catfish (*Ictalurus punctatus*), respectively [51,52], it is assumed that the growth of olive flounder was not negatively affected by slightly low methionine content in the experimental feeds due to the presence of high amounts of cysteine (0.59–0.67%) in this experiment.

Long-chain n-3 HUFA including DHA and EPA are considered indispensable FAs for appropriate growth and development of olive flounder [53]. They must be supplied through diets because farmed fish have limited or no capacity to synthesize them in their bodies [54]. The ∑n-3 HUFA in the Con, DJ40, and DJ50 diets met ∑n-3 HUFA requirements in the feed of olive flounder (5.80–7.25% of total FA) [40]. This likely explains why the fish fed DJ0, DJ10, DJ20, and DJ30 diets showed reduced growth performance compared to fish fed the Con diet in this experiment. Higher ∑SFA, and ∑n-3 HUFA, but lower ∑MUFA content in the whole body of fish fed the Con diet were attained based on FA profiles of the experimental feeds in this experiment. These findings are supported by other studies [16,48] showing that dietary FA profiles were mirrored in the whole-body FA profiles of fish.

Plasma measurements are strongly correlated with the health, nutritional status, and environmental condition of fish and can reveal the physiological and metabolic status of fish [55]. No distinctive changes in plasma parameters in this study indicates that olive flounder were in similar nutritional and physiological conditions. Similarly, incorporated protein hydrolysates in feeds did not affect the plasma parameters of red sea bream [56] or olive flounder, except for ALT [46].

Serum SOD and lysozyme are important defense enzymes that play significant roles in detoxifying free radicals during oxidative stress conditions and in lysing the bacterial cell wall during bacterial invasion, respectively [57]. In this experiment, no significant differences in serum SOD or lysozyme activity of fish were found. This is consistent with previous studies, where no significant difference in serum SOD or lysozyme activity in olive flounder were observed following dietary replacement of fermented tuna by-product meal [58], chicken by-product meal [15], and meat meal [16] for FM. However, in contradiction to this study, Tharaka et al. [45] observed an improvement in serum SOD and lysozyme activity in olive flounder fed low-FM diets supplemented with Antarctic krill (*Euphausia superba*) meal, probably due to the presence of chitin, phospholipid, and astaxanthin, which have an immunostimulatory effect.

The chemical composition and AA profile of the whole-body olive flounder were not affected by dietary treatments in the present experiment. Likewise, dietary substitution of fish meal with chicken by-product meal up to 50% level did not alter the carcass composition or AA profile of olive flounder [15], and total substitution of FM with poultry by-product meal did not alter the muscle AA profile of juvenile gilthead seabream (*Sparus aurata*) [59], except tyrosine and threonine. The incorporation of protein hydrolysates in low-FM diets [46] or different AA patterns in the experimental feeds [36] did not change the proximate composition or whole-body AA profile of olive flounder. Likewise, replacing FM with animal by-product meal [48] and meat meal produced from pig [16] caused no changes in the whole-body chemical composition or AA profile of olive flounder either. Nevertheless, there are also some contradicting studies, where FM replacements with animal and plant proteins affected whole-body proximate composition [14,17] and AA profiles [60,61] of olive flounder.

## 5. Conclusions

Inclusion of JMM in low-FM diets replacing 50% FM with DBM improved weight gain, SGR, and feed consumption of olive flounder. Comparable weight gain, SGR, and feed intake were obtained in fish fed the DJ40 and DJ50 diets compared to fish fed the Con (60% FM-based) diet. Furthermore, the DJ50 diet led to the highest EPI. Therefore, inclusion of 50% JMM in low-FM feed replacing 50% FM with DBM is the most recommendable treatment based on growth and feed intake of olive flounder and economic return to the farmer.

## Figures and Tables

**Table 1:** Ingredients and chemical composition of the experimental diets (%, dry matter basis).

	Experimental Diets						
	Con	DJ0	DJ10	DJ20	DJ30	DJ40	DJ50
Ingredients (%, DM)							
Fish meal (FM) ^a^	60.00	30.00	24.00	18.00	12.00	6.00	0.00
Duck by-product meal (DBM) ^b^	-	34.70	34.70	34.70	34.70	34.70	34.70
Jack mackerel meal (JMM) ^c^	-	0.00	6.00	12.00	18.00	24.00	30.00
Fermented soybean meal	10.00	10.00	10.00	10.00	10.00	10.00	10.00
Wheat flower	22.50	18.80	18.70	18.65	18.60	18.55	18.50
Fish oil	2.50	2.50	2.50	2.50	2.50	2.50	2.50
Soybean oil	2.50	1.50	1.60	1.65	1.70	1.75	1.80
Vitamin premix ^d^	1.00	1.00	1.00	1.00	1.00	1.00	1.00
Mineral premix ^e^	1.00	1.00	1.00	1.00	1.00	1.00	1.00
Choline	0.50	0.50	0.50	0.50	0.50	0.50	0.50
Nutrients (%, DM)							
Dry matter	97.79	97.29	98.00	98.47	98.30	97.98	97.73
Crude protein	52.52	52.18	52.82	51.04	51.14	51.42	51.35
Crude lipid	13.61	14.17	13.82	13.14	14.33	13.96	13.60
Ash	12.13	12.92	13.13	12.06	12.29	12.94	12.11

^a^ Fish meal (FM) (crude protein: 70.1%, crude lipid: 8.5%, ash: 16.6%) composed of sardine meal and anchovy meal at the ratio of 1:1 was imported from Peru (USD 2.23/kg FM, USD 1 = KRW 1232 (South Korean currency)). ^b^ Duck by-product meal (DBM) (crude protein: 61.5%, crude lipid: 11.4%, ash: 19.4%) was purchased from Gbiotech (Seongnam, Gyeonggi-do, South Korea) (USD 0.60/kg DBM). ^c^ Jack mackerel meal (JMM) (crude protein: 72.2%, crude lipid: 9.9%, ash: 14.3%) was imported from Chile (USD 2.67/kg JMM). ^d^ Vitamin premix (g/kg mix): L-ascorbic acid, 121.2; DL-α-tocopheryl acetate, 75.7; thiamin hydrochloride, 2.7; riboflavin, 9.1; pyridoxine hydrochloride, 1.8; niacin, 36.4; Ca-D-pantothenate, 12.7; myo-inositol, 181.8; D-biotin, 5.45; folic acid, 0.68; p-aminobenzoic acid, 18.2; menadione, 1.8; retinyl acetate, 0.73; cholecalciferol, 0.003; cyanocobalamin, 0.003. ^e^ Mineral premix (g/kg mix): MgSO_4_·7H_2_O, 80.0; NaH_2_PO_4_·2H_2_O, 370.0; KCl, 130.0; ferric citrate, 40.0; ZnSO_4_·7H_2_O, 20.0; Ca-lactate, 356.5; CuCl, 0.2; AlCl_3_·6H_2_O, 0.15; KI, 0.2; Na_2_Se_2_O_3_, 0.01; MnSO_4_·H_2_O, 2.0; CoCl_2_·6H_2_O, 1.0.

**Table 2:** Amino acid profiles (% of the diet) of feed ingredients and the experimental diets.

	Ingredients				Experimental Diets						
	FM	JMM	DBM	Requirement of Olive Flounder	Con	DJ0	DJ10	DJ20	DJ30	DJ40	DJ50
Essential amino acids (EAA, %)											
Arginine	3.51	3.90	3.98	2.04–2.10 ^a^	2.66	2.90	2.93	2.97	3.00	3.05	3.13
Histidine	1.50	2.83	1.15		1.05	1.03	1.09	1.19	1.24	1.36	1.47
Isoleucine	2.47	2.76	2.01		1.78	1.65	1.68	1.71	1.76	1.79	1.84
Leucine	4.50	4.83	3.74		3.42	3.27	3.30	3.34	3.39	3.41	3.44
Lysine	4.82	5.31	3.44	1.55–2.16 ^b^	3.33	3.05	3.09	3.14	3.17	3.21	3.30
Methionine	1.75	1.74	1.17	1.44–1.49 ^c^	1.15	1.04	1.06	1.06	1.07	1.07	1.11
Phenylalanine	2.42	2.60	2.05		1.95	1.85	1.82	1.86	1.83	1.89	1.94
Threonine	2.69	2.88	2.12	1.03 ^d^	2.04	1.94	1.96	1.96	1.99	1.99	2.05
Tryptophan	0.49	0.57	0.42		0.34	0.26	0.32	0.35	0.37	0.40	0.41
Valine	2.96	3.22	2.41		2.11	1.88	1.94	1.96	2.05	2.11	2.15
∑EAA ^e^	27.11	30.64	22.49		19.83	18.87	19.19	19.54	19.87	20.28	20.84
Non-essential amino acid (NEAA, %)											
Alanine	4.04	4.29	4.29		2.89	3.05	3.07	3.11	3.11	3.14	3.17
Aspartic acid	5.58	5.91	4.47		4.32	4.07	4.09	4.14	4.19	4.26	4.29
Cysteine	0.80	0.83	0.63	0.06 ^c^	0.63	0.59	0.62	0.62	0.64	0.64	0.67
Glutamic acid	8.08	8.34	7.67		7.05	6.96	6.98	7.06	7.11	7.18	7.24
Glycine	3.94	4.29	6.28		2.83	3.58	3.63	3.66	3.69	3.71	3.81
Proline	2.65	2.98	4.18		2.34	2.72	2.76	2.81	2.85	2.87	2.90
Serine	2.46	2.59	1.98		2.03	1.94	1.95	1.99	1.99	2.00	2.02
Tyrosine	1.53	1.71	1.47		1.11	1.10	1.10	1.13	1.15	1.28	1.23
∑NEAA ^f^	29.08	30.94	30.97		23.2	24.01	24.2	24.52	24.73	25.08	25.33

^a^ Arginine, ^b^ lysine, ^c^ methionine, and ^d^ threonine requirements were obtained from the studies of Alam et al. [36], Forster and Ogata [37], Alam et al. [38], and Hasanthi et al. [39], respectively; ^e^ ∑EAA: total content of essential amino acids; ^f^ ∑NEAA: total content of non-essential amino acids; FM: fish meal; JMM: jack mackerel meal; DBM: duck by-product meal; Con: 60% FM; DJ0–DJ50: 50% DBM with 0% to 50% JMM.

**Table 3:** Fatty acid profiles (% of total fatty acids) of feed ingredients and the experimental diets.

	Ingredients				Experimental Diets						
Fatty Acid (%)	FM	JMM	DBM	Requirement of Olive Flounder	Con	DJ0	DJ10	DJ20	DJ30	DJ40	DJ50
C12:0	0.08	0.06	0.05		0.05	0.05	0.05	0.05	0.05	0.04	0.04
C14:0	4.20	3.29	0.59		3.43	2.00	1.98	1.80	1.74	1.68	1.69
C16:0	23.10	21.05	22.93		17.56	17.66	17.41	16.86	16.73	16.5	16.13
C18:0	8.05	7.58	7.15		4.24	3.87	3.38	3.63	3.62	3.57	3.42
C20:0	0.10	0.10	0.07		0.19	0.12	0.13	0.12	0.12	0.11	0.13
C22:0	0.30	0.16	0.24		0.24	0.21	0.20	0.16	0.15	0.13	0.12
C24:0	0.68	0.50	0.28		0.62	0.38	0.38	0.37	0.39	0.34	0.35
∑SFA ^a^	36.51	32.74	31.31		26.33	24.29	23.53	22.99	22.80	22.37	21.88
C14:1n-5	0.23	0.15	0.12		0.08	0.05	0.06	0.09	0.05	0.09	0.09
C16:1n-7	5.47	4.50	4.28		3.82	3.62	3.56	3.51	3.47	3.41	3.34
C18:1n-9	24.08	24.33	48.30		26.78	37.29	37.30	37.39	37.47	37.51	37.65
C20:1n-9	1.01	1.54	0.45		0.86	0.58	0.64	0.66	0.69	0.73	0.77
C22:1n-9	0.19	0.16	0.23		0.22	0.26	0.27	0.22	0.26	0.29	0.28
C24:1n-9	2.69	4.00	0.09		1.20	0.64	0.66	0.71	0.76	0.77	0.82
∑MUFA ^b^	33.67	34.68	53.47		32.96	42.44	42.49	42.58	42.70	42.80	42.95
C18:2n-6	1.89	1.33	12.63		23.16	24.01	24.41	25.31	25.34	25.33	24.96
C18:3n-3	0.70	0.51	0.47		2.79	1.99	1.92	1.90	1.87	1.86	1.86
C18:3n-6	0.30	0.16	0.10		0.50	0.34	0.35	0.35	0.26	0.37	0.34
C20:2n-6	0.07	0.24	0.08		0.22	0.09	0.07	0.14	0.08	0.09	0.06
C20:3n-3	0.17	0.09	0.05		0.00	0.00	0.00	0.02	0.00	0.02	0.00
C20:3n-6	0.08	0.00	0.20		0.05	0.08	0.08	0.09	0.08	0.08	0.06
C20:4n-6	2.44	1.74	1.23		1.22	0.75	0.82	0.84	0.86	0.87	0.89
C20:5n-3	7.06	10.98	0.10		7.3	3.03	3.11	3.23	3.29	3.41	3.57
C22:6n-3	12.18	14.71	0.05		4.25	2.17	2.28	2.36	2.44	2.66	2.79
∑n-3 HUFA ^c^	19.41	25.78	0.20	5.80–7.25 ^d^	11.55	5.20	5.39	5.61	5.73	6.09	6.36
Unknown	4.93	2.82	0.31		1.22	0.76	0.84	0.26	0.26	0.32	0.64

^a^ ∑SFA: total content of saturated fatty acids; ^b^ ∑MUFA: total content of monounsaturated fatty acids; ^c^ ∑n-3 HUFA: total content of n-3 highly unsaturated fatty acids; ^d^ ∑n-3 HUFA was obtained from Kim and Lee’s [40] study; FM: fish meal; JMM: jack mackerel meal; DBM: duck by-product meal; Con: 60% FM; DJ0–DJ50: 50% DBM with 0% to 50% JMM.

**Table 4:** Survival (%), feed efficiency (FE), protein efficiency ratio (PER), protein retention (PR), condition factor (K), viscerosomatic index (VSI), and hepatosomatic index (HSI) of olive flounder fed the experimental diets for 56 days.

	Experimental Diets									Orthogonal Polynomial Contrast			Regression		
	Con	DJ0	DJ10	DJ20	DJ30	DJ40	DJ50	SEM	*p*-Value	Linear	Quadratic	Cubic	Model	*p*-Value	Adj. R^2^
Initial weight (g/fish)	20.35	20.27	20.16	20.29	20.19	20.35	20.27	0.03							
Final weight (g/fish)	76.47	70.5	70.72	71.44	72.97	75.09	75.83	0.53	0.001	0.001	0.073	0.123	L	0.001	0.850
Survival (%)	94.67	94.67	97.33	96.00	96.00	96.00	97.33	0.68	0.893	0.611	0.943	0.414	NR		
FCR ^a^	0.96 ^b^	1.00 ^a^	1.00 ^a^	1.02 ^a^	1.01 ^a^	0.99 ^ab^	0.98 ^ab^	0.01	0.003	0.050	0.026	0.799	Q	0.012	0.368
PER ^b^	1.99 ^ab^	1.91 ^bc^	1.89 ^c^	1.93 ^abc^	1.93 ^abc^	1.96 ^abc^	1.99 ^a^	0.01	0.005	0.001	0.222	0.733	L	0.001	0.533
PR (%) ^c^	33.87	31.23	34.03	31.33	31.50	30.57	33.64	0.43	0.142	0.756	0.337	0.015	NR		
K (g/cm^3^) ^d^	0.86	0.90	0.86	0.84	0.84	0.87	0.91	0.01	0.201	0.709	0.008	0.987	NR		
VSI (%) ^e^	4.26	4.63	4.14	4.43	4.40	4.57	4.36	0.06	0.242	0.879	0.495	0.021	NR		
HSI (%) ^f^	1.20	1.43	1.09	1.34	1.28	1.22	1.10	0.05	0.290	0.175	0.895	0.157	NR		

Values (mean of triplicate) in the same column sharing the same superscript letter are not significantly different (*p* > 0.05); Con: 60% FM; DJ0–DJ50: 50% DBM with 0% to 50% JMM. Abbreviations: SEM, pooled standard error of treatment means; Adj. R^2^, adjusted R squared; L, linear; Q, quadratic; C, cubic; NR, no relationship. ^a^ Feed conversion ratio (FCR) = feed supplied/weight gain of fish; ^b^ protein efficiency ratio (PER) = weight gain of fish/protein supplied; ^c^ protein retention (PR, %) = (final body protein − initial body protein) × 100/protein supplied; ^d^ condition factor (K, g/cm^3^) = body weight of fish × 100/total length of fish (cm)^3^; ^e^ viscerosomatic index (VSI, %) = viscera weight of fish × 100/body weight of fish; ^f^ hepatosomatic index (HSI, %) = liver weight of fish × 100/body weight of fish. Inclusion levels of JMM in diets were used as the independent variables, and final weight, survival, FCR, PER, PR, K, VSI, and HSI were used as dependent variables in regression analysis.

**Table 5:** Chemical composition (%, wet weight) of whole-body olive flounder at the end of the 56-day feeding trial.

	Experimental Diets									Orthogonal Polynomial Contrast			Regression		
	Con	DJ0	DJ10	DJ20	DJ30	DJ40	DJ50	SEM	*p*-Value	Linear	Quadratic	Cubic	Model	*p*-Value	Adj. R^2^
Moisture	75.20	75.03	74.63	75.63	75.10	75.00	75.13	0.20	0.904	0.817	0.732	0.993	NR		
Crude protein	16.87	16.33	17.47	16.6	16.63	16.10	16.63	0.15	0.271	0.377	0.597	0.031	NR		
Crude lipid	2.87	3.63	2.80	2.93	2.63	2.93	3.70	0.17	0.435	0.689	0.246	0.555	NR		
Ash	3.67	3.27	4.47	4.33	3.97	3.57	3.87	0.14	0.357	0.979	0.069	0.021	NR		

Values (mean of triplicate) in the same column sharing the same superscript letter are not significantly different (*p* > 0.05). Con: 60% FM; DJ0–DJ50: 50% DBM with 0% to 50% JMM. Abbreviations: SEM, pooled standard error of treatment means; Adj. R^2^, adjusted R square; L, linear; Q, quadratic; C, cubic; NR, no relationship. Inclusion levels of JMM in diets were used as the independent variables and moisture, crude protein, crude lipid, and ash content were used as the dependent variable in regression analysis.

**Table 6:** Plasma and serum parameters of olive flounder at the end of the 56-day feeding trial.

	Experimental Diets									Orthogonal Polynomial Contrast			Regression		
	Con	DJ0	DJ10	DJ20	DJ30	DJ40	DJ50	SEM	*p*-Value	Linear	Quadratic	Cubic	Model	*p*-Value	Adj. R^2^
Plasma parameters															
AST (U/L)	9.67	10.67	9.00	10.67	9.00	9.00	8.33	0.41	0.534	0.413	0.454	0.765	NR		
ALT (U/L)	5.00	5.33	6.00	5.66	5.66	5.00	4.67	0.31	0.942	0.211	0.983	0.869	NR		
ALP (U/L)	70.00	70.33	71.66	71.00	71.33	73.67	72.67	0.62	0.699	0.686	0.115	0.705	NR		
T-BIL (mg/dL)	0.17	0.23	0.17	0.20	0.13	0.23	0.23	0.02	0.386	0.550	0.770	0.770	NR		
T-CHO (mg/dL)	181.33	181.0	179.0	180.67	181.33	181.0	181.67	0.70	0.968	0.642	0.313	0.583	NR		
TG (mg/dL)	438.33	443.0	440.33	441.0	442.67	441.22	443.33	0.61	0.395	0.582	0.446	0.763	NR		
TP (g/dL)	1.93	2.13	1.93	2.30	1.73	2.23	2.33	0.11	0.655	0.897	0.310	0.701	NR		
ALB (g/dL)	0.47	0.63	0.43	0.53	0.43	0.53	0.57	0.04	0.862	0.133	0.896	0.119	NR		
Serum parameters															
SOD (ng/mL)	2.76	3.12	2.58	2.67	2.70	2.57	2.83	0.01	0.815	0.524	0.332	0.683	NR		
Lysozyme (U/mL)	546.67	445.0	455.00	296.67	348.33	406.67	431.67	43.47	0.878	0.876	0.409	0.969	NR		

Values (mean of triplicate) in the same column sharing the same superscript letter are not significantly different (*p* > 0.05). Con: 60% FM; DJ0–DJ50: 50% DBM with 0% to 50% JMM. Abbreviations: SEM, pooled standard error of treatment means; Adj. R^2^, adjusted R square; L, linear; Q, quadratic; C, cubic; NR, no relationship. Inclusion levels of JMM in diets were used as the independent variables and plasma and serum parameters were used as the dependent variable in regression analysis.

**Table 7:** Amino acid profiles (% of wet weight) of the whole-body of olive flounder fed the experimental diets for 56 days.

	Experimental Diets									Orthogonal Polynomial Contrast			Regression		
	Con	DJ0	DJ10	DJ20	DJ30	DJ40	DJ50	SEM	*p*-Value	Linear	Quadratic	Cubic	Model	*p*-Value	Adj. R^2^
Essential amino acid (%)															
Arginine	1.06	0.94	1.09	1.09	1.04	1.05	1.03	0.02	0.481	0.530	0.098	0.195	NR		
Histidine	0.33	0.32	0.35	0.35	0.33	0.27	0.34	0.01	0.200	0.368	0.860	0.072	NR		
Isoleucine	0.65	0.63	0.70	0.68	0.65	0.64	0.65	0.01	0.435	0.396	0.287	0.072	NR		
Leucine	1.15	1.12	1.25	1.22	1.18	1.23	1.16	0.01	0.089	0.666	0.036	0.235	NR		
Lysine	1.33	1.31	1.44	1.41	1.36	1.27	1.35	0.02	0.179	0.306	0.200	0.016	NR		
Methionine	0.45	0.43	0.49	0.48	0.47	0.45	0.43	0.01	0.335	0.384	0.035	0.266	NR		
Phenylalanine	0.61	0.59	0.65	0.66	0.63	0.62	0.62	0.01	0.530	0.844	0.104	0.178	NR		
Threonine	0.72	0.69	0.76	0.75	0.72	0.74	0.72	0.01	0.538	0.746	0.235	0.193	NR		
Tryptophan	0.12	0.11	0.12	0.10	0.11	0.11	0.10	0.01	0.996	0.903	0.999	0.939	NR		
Valine	0.75	0.73	0.80	0.79	0.76	0.75	0.75	0.01	0.442	0.819	0.172	0.083	NR		
Non-essential amino acids (%)															
Alanine	1.14	1.03	1.17	1.19	1.13	1.22	1.10	0.02	0.266	0.249	0.039	0.728	NR		
Aspartic acid	1.15	1.46	1.60	1.59	1.53	1.53	1.52	0.02	0.508	0.925	0.117	0.105	NR		
Cysteine	0.21	0.21	0.23	0.23	0.23	0.24	0.21	0.01	0.843	0.730	0.207	0.774	NR		
Glutamic acid	2.30	2.19	2.42	2.38	2.27	2.45	2.30	0.04	0.387	0.480	0.231	0.497	NR		
Glycine	1.38	1.11	1.33	1.38	1.27	1.26	1.26	0.03	0.411	0.521	0.099	0.130	NR		
Proline	0.83	0.70	0.83	0.84	0.80	0.86	0.79	0.02	0.373	0.293	0.081	0.506	NR		
Serine	0.77	0.72	0.79	0.79	0.76	0.75	0.76	0.02	0.956	0.854	0.432	0.371	NR		
Tyrosine	0.40	0.39	0.44	0.43	0.40	0.38	0.40	0.01	0.910	0.561	0.601	0.310	NR		

Values (mean of triplicate) in the same row sharing the same superscript letter are not significantly different (*p* > 0.05). Con: 60% FM; DJ0–DJ50: 50% DBM with 0% to 50% JMM. Abbreviations: SEM, pooled standard error of treatment means; Adj. R^2^, adjusted R square; L, linear; Q, quadratic; C, cubic; NR, no relationship. Inclusion levels of JMM in diets were used as the independent variables and amino acid profiles were used as the dependent variable in regression analysis.

**Table 8:** Fatty acid profiles (% of total fatty acids) of the whole body of olive flounder fed the experimental diets for 56 days.

	Experimental Diets									Orthogonal Polynomial Contrast			Regression		
	Con	DJ0	DJ10	DJ20	DJ30	DJ40	DJ50	SEM	*p*-Value	Linear	Quadratic	Cubic	Model	*p*-Value	Adj. R^2^
C12:0	0.04	0.04	0.03	0.04	0.04	0.04	0.04	0.00	0.933	0.671	0.471	0.575	NR		
C14:0	3.74 ^a^	2.46 ^b^	2.42 ^b^	2.35 ^b^	2.31 ^b^	2.24 ^b^	2.21 ^b^	0.03	0.001	0.005	0.932	0.819	L	0.001	0.468
C16:0	17.87 ^ab^	18.25 ^a^	18.03 ^ab^	17.78 ^ab^	17.79 ^ab^	17.58 ^b^	17.54 ^b^	0.07	0.006	0.001	0.323	0.727	L	0.001	0.643
C18:0	3.76 ^a^	3.56 ^ab^	3.44 ^abc^	3.35 ^bc^	3.46 ^abc^	3.28 ^bc^	3.08 ^c^	0.05	0.001	0.002	0.341	0.162	L	0.001	0.460
C20:0	0.12	0.10	0.10	0.10	0.10	0.09	0.10	0.00	0.068	0.470	0.720	0.210	NR		
C22:0	0.55	0.60	0.58	0.61	0.63	0.59	0.63	0.01	0.719	0.560	0.961	0.868	NR		
C24:0	0.80 ^a^	0.49 ^b^	0.47 ^b^	0.49 ^b^	0.43 ^b^	0.42 ^b^	0.37 ^b^	0.02	0.001	0.025	0.442	0.978	L	0.013	0.288
∑SFA ^a^	26.88 ^a^	25.50 ^b^	25.07 ^c^	24.72 ^d^	24.76 ^d^	24.24 ^e^	23.98 ^f^	0.12	0.001	0.002	0.813	0.062	L	0.001	0.927
C14:1n-5	0.15	0.11	0.10	0.09	0.10	0.11	0.09	0.00	0.051	0.343	0.916	0.290	NR		
C16:1n-7	5.09	4.86	4.90	4.97	5.05	5.10	5.07	0.03	0.149	0.001	0.618	0.998	NR		
C18:1n-9	21.57 ^e^	33.14 ^d^	33.20 ^cd^	33.32 ^bcd^	33.46 ^abc^	33.56 ^ab^	33.66 ^a^	0.92	0.001	0.001	0.886	0.384	L	0.001	0.906
C20:1n-9	2.13 ^a^	1.75 ^b^	1.80 ^b^	1.75 ^b^	1.74 ^b^	1.75 ^b^	1.72 ^b^	0.02	0.001	0.582	0.738	0.848	NR		
C22:1n-9	0.28	0.25	0.27	0.27	0.26	0.25	0.27	0.01	0.742	0.817	0.818	0.146	NR		
C24:1n-9	1.96 ^a^	1.34 ^e^	1.47 ^de^	1.51 ^cde^	1.66 ^bcd^	1.71 ^bc^	1.87 ^ab^	0.04	0.001	0.001	0.711	0.562	L	0.001	0.886
∑MUFA ^b^	31.17 ^e^	41.44 ^d^	41.75 ^cd^	41.92 ^bc^	42.27 ^ab^	42.47 ^a^	42.68 ^a^	0.85	0.001	0.001	0.641	0.842	L	0.003	0.891
C18:2n-6	15.74 ^e^	16.25 ^ad^	16.50 ^acd^	16.62 ^bc^	16.84 ^b^	16.75 ^bc^	17.16 ^a^	0.07	0.001	0.001	0.942	0.023	L	0.001	0.834
C18:3n-3	1.78 ^a^	1.12 ^b^	1.09 ^b^	1.05 ^b^	1.03 ^b^	1.00 ^b^	1.00 ^b^	0.02	0.001	0.025	0.618	0.834	L	0.001	0.305
C18:3n-6	0.40	0.32	0.37	0.36	0.33	0.33	0.36	0.01	0.320	0.657	0.726	0.105	NR		
C20:2n-6	0.16 ^a^	0.12 ^a^	0.07 ^b^	0.09 ^b^	0.07 ^b^	0.09 ^b^	0.08 ^b^	0.01	0.002	0.094	0.040	0.188	NR		
C20:3n-3	0.03	0.03	0.04	0.04	0.04	0.04	0.02	0.00	0.362	0.322	0.100	0.779	NR		
C20:3n-6	0.03 ^b^	0.10 ^a^	0.07 ^ab^	0.08 ^ab^	0.08 ^ab^	0.08 ^ab^	0.09 ^a^	0.00	0.003	0.468	0.030	0.472	NR		
C20:4n-6	3.88 ^a^	2.28 ^c^	2.32 ^c^	2.40 ^c^	2.48 ^bc^	2.56 ^b^	2.61 ^b^	0.03	0.001	0.001	0.909	0.601	NR	0.001	0.764
C20:5n-3	6.11 ^a^	3.01 ^e^	3.61 ^d^	3.70 ^d^	3.90 ^c^	4.14 ^b^	4.25 ^b^	0.10	0.001	0.000	0.001	0.001	C	0.001	0.963
C22:6n-3	10.66 ^a^	4.65 ^d^	5.07 ^c^	5.05 ^c^	5.14 ^c^	5.30 ^b^	5.42 ^b^	0.43	0.001	0.000	0.011	0.001	C	0.001	0.948
∑n-3 HUFA ^c^	16.80 ^a^	7.69 ^f^	8.72 ^e^	8.79 ^e^	9.08 ^d^	9.48 ^c^	9.70 ^b^	0.63	0.001	0.000	0.001	0.001	C	0.001	0.964
Unknown	3.15	5.16	4.02	3.96	3.06	3.00	2.34	0.20							

Values (mean of triplicate) in the same row sharing the same superscript letter are not significantly different (*p >* 0.05). Con: 60% FM; DJ0–DJ50: 50% DBM with 0% to 50% JMM. Abbreviations: SEM, pooled standard error of treatment means; Adj. R^2^, adjusted R square; L, linear; Q, quadratic; C, cubic; NR, no relationship. ^a^ ∑SFA: Total content of saturated fatty acids; ^b^ ∑MUFA: Total content of monounsaturated fatty acids; ^c^ ∑n-3 HUFA: Total content of n-3 highly unsaturated fatty acids. Inclusion levels of JMM in diets were used as the independent variables and fatty acid profiles were used as the dependent variable in regression analysis.

**Table 9:** Effect of dietary replacement of fish meal (FM) with duck by-product meal (DBM) supplemented with the graded levels of jack mackerel meal (JMM) on economic parameters of the study.

	Experimental Diets									Orthogonal Polynomial Contrast			Regression		
	Con	DJ0	DJ10	DJ20	DJ30	DJ40	DJ50	SEM	*p*-Value	Linear	Quadratic	Cubic	Model	*p*-Value	Adj. R^2^
Diet price (USD/kg)	1.80	1.30	1.33	1.36	1.38	1.41	1.44								
ECR (USD/kg) ^a^	1.73 ^a^	1.31 ^d^	1.33 ^cd^	1.38 ^bc^	1.40 ^b^	1.40 ^b^	1.41 ^b^	0.03	0.001	0.001	0.030	0.743	Q	0.001	0.732
EPI (USD/fish) ^b^	0.85 ^ab^	0.81 ^c^	0.81 ^c^	0.82 ^c^	0.83 ^bc^	0.86 ^a^	0.87 ^a^	0.01	0.001	0.000	0.045	0.131	L	0.001	0.812

Values (mean of triplicate) in the same row sharing the same superscript letter are not significantly different (*p* > 0.05). Con: 60% FM; DJ0–DJ50: 50% DBM with 0% to 50% JMM. ^a^ Economic conversion ratio (ECR, USD/kg) = feed consumption of fish (kg) × feed cost (USD/kg)/weight gain (kg); ^b^ Economic profit index (EPI, USD/fish) = (final weight of fish (kg/fish) × selling price of fish (USD/kg)) – (feed consumption of fish (kg) × diet price (USD/kg)). Inclusion levels of JMM in diets were used as the independent variables and ECR and EPI were used as the dependent variables in regression analysis.

## Data Availability

Data supporting the conclusion of this article will be made available on request from the authors.
